# Awareness and current knowledge of breast cancer

**DOI:** 10.1186/s40659-017-0140-9

**Published:** 2017-10-02

**Authors:** Muhammad Akram, Mehwish Iqbal, Muhammad Daniyal, Asmat Ullah Khan

**Affiliations:** 1 Department of Eastern Medicine and Surgery, Directorate of Medical Sciences, GC University Faisalabad, Old Campus, Allam Iqbal Road, Faisalabad, 38000 Pakistan; 2 Faculty of Eastern Medicine, Hamdard University Karachi, Main Campus, Sharea Madinat al-Hikmah, Mohammad Bin Qasim Avenue, Karachi, 74600 Sindh Pakistan; 3 Laboratory of Neuroanatomy & Neuropsychobiology, Department of Pharmacology, RibeirãoPreto Medical School of the University of São Paulo, AV. Bandeirantes, 3900, RibeirãoPreto, 14049-900 São Paulo, Brazil; 4 Department of Eastern Medicine and Surgery, School of Medical and Health Sciences, University of Poonch Rawalakot, Hajira Road, Shamsabad, Rawalakot, 12350 Azad Jammu and Kashmir Pakistan

**Keywords:** Breast cancer, Chemotherapy, Gene therapy, Stem cell therapy

## Abstract

Breast cancer remains a worldwide public health dilemma and is currently the most common tumour in the globe. Awareness of breast cancer, public attentiveness, and advancement in breast imaging has made a positive impact on recognition and screening of breast cancer. Breast cancer is life-threatening disease in females and the leading cause of mortality among women population. For the previous two decades, studies related to the breast cancer has guided to astonishing advancement in our understanding of the breast cancer, resulting in further proficient treatments. Amongst all the malignant diseases, breast cancer is considered as one of the leading cause of death in post menopausal women accounting for 23% of all cancer deaths. It is a global issue now, but still it is diagnosed in their advanced stages due to the negligence of women regarding the self inspection and clinical examination of the breast. This review addresses anatomy of the breast, risk factors, epidemiology of breast cancer, pathogenesis of breast cancer, stages of breast cancer, diagnostic investigations and treatment including chemotherapy, surgery, targeted therapies, hormone replacement therapy, radiation therapy, complementary therapies, gene therapy and stem-cell therapy etc for breast cancer.

## Background

Breast cancer is the most common cancer and also the primary cause of mortality due to cancer in female around the World. About 1.38 million new breast cancer cases were diagnosed in 2008 with almost 50% of all breast cancer patients and approximately 60% of deaths occurring in developing countries. There is a huge difference in breast cancer survival rates worldwide, with an estimated 5-year survival of 80% in developed countries to below 40% for developing countries [1]. Developing countries face resource and infrastructure constraints that challenge the objective of improving breast cancer outcomes by timely recognition, diagnosis and management [2]. In developed countries like the United States, about 232,340 female will be diagnosed and death of 39,620 female will occur due to breast cancer in 2013 [3]. The lifetime risk of developing breast cancer in an American female is 12.38% [3]. The significant decline in morality due to breast cancer in the United States from 1975 to 2000 is attributed to constant enhancement in both screening mammography and management [4]. According to the World Health Organization (WHO), enhancing breast cancer outcome and survival by early detection remains the foundation of breast cancer regulations. Different modern medicines are prescribed to treat breast cancer. Medical therapy of breast cancer with antiestrogens such as raloxifene or tamoxifen might avoid breast cancer in individuals who are at increased possibility of developing it [5]. Surgery of both breasts is an added preventative measure in some increased probability of developing cancer in female. In patients who have been identified with breast tumor, different strategies of management are used such as targeted therapy, hormonal therapy, radiation therapy, surgery and chemotherapy. In individuals with distant metastasis, managements are typically aimed at enhancing life quality and survival rate [6]. The unpleasant side effects of breast cancer treatment are one of the most motivating factors to find some alternative methods. The use of herbs for treating the patients having breast cancer is considered a natural alternative, because some plants may contain properties that naturally have the ability to treat breast cancer [7–11].

## Epidemiology

Currently, one in twelve females in Britain between age of 1 and 85 years gets breast cancer. With one million new cases of cancers reported in the World, breast cancer is common in females and comprises 18% of all women cancer. Incidence of breast cancer is predicted to increase to 85 per 100,000 women by 2021 [12]. In 2012, 1.67 million new cases of breast cancer were diagnosed that is 25% of all cancers among women. Ferlay et al. [13] stated that 883,000 cases are in less developed countries and 794,000 in most developed countries. According to the data, 145.2 women in Belgium and 66.3 in Poland between 100,000 suffer from breast cancer [14]. Incidence of breast cancer in the United States is one out of eight women and In Asia one woman suffers from breast cancer out of 35. In Iran, there are 10 cases in 100,000 populations and 7000 new cases have been reported annually [15]. Prevalence of breast cancer is increasing in Pakistan [16–18]. Breast cancer is found mostly in highly populated areas of South Asian developing counties [19, 20]. Breast cancers in males have been detected in Northern areas of Pakistan [21]. Yang et al. [22] stated that new cases of breast cancer in China were 168,013 in 2005 and 121,269 in 2000.

## Anatomy of breast

Both males and females have breasts [23]. The breast is made up of fatty tissue called adipose tissue [24]. The female’s breasts usually contain more glandular tissue than that of the males [25]. Female breasts contain 12–20 lobes which are further divided into smaller lobules [26]. These lobes and lobules are connected via milk ducts. The adipose tissue of the breast is supplied by a network of nerves, blood vessels, lymph vessels, lymph nodes, and is also composed of fibrous connective tissue and ligaments [27]. The female breast is designed to provide optimal nourishment for babies and to provide sexual pleasure for the female herself. The breasts are glandular organs that are very sensitive to hormonal changes in the body [28]. They adopt cyclic changes in synchrony with the menstrual cycle. They are closely associated with the genital system of females. Nipple stimulation enhances secretion of prolactin from the pituitary gland. This hormone also affects the uterus and can cause contractions. Lymph node draining the breast tissues is also found in the armpits. After a female has had a baby and her milk comes in, mother may develop striking swelling under arms from engorgement of the breast tissue in that region. Breasts come in all sizes and shapes, as do nipples. Most female possess one breast that slightly smaller than the other [29]. The epidermis of the areola and nipple is very much pigmented and to some extent wrinkled, and the nipple skin contains several apocrine and sebaceous sweat glands and somewhat small hair. The 15–25 milk ducts go into the base of the nipple, wherever they expand to synthesize the milk sinuses. These milk ducts functions as the carriers of milk towards the nipples [30]. Slightly under the surface of nipple, these sinuses end in cone-shaped ampullae. The spherical areola is present around the nipple and is between 15 and 60 mm in diameter. Sebaceous glands, sweat glands and lanugo hairs are present on its skin, Montgomery’s glands, are big, modified sebaceous glands with tiny milk ducts that open into Morgagni’s tubercles in the areola epidermis. Deep in the nipple and areola, several smooth muscle fibers are set circularly and radially in the dense connective tissue and longitudinally alongside the lactiferous ducts that lengthen up into the nipple. These muscle fibers are cause emptying of milk sinuses, nipple erection and contraction of areola. The greater part of the breast parenchyma expand inferiorly from the point of the 2nd or 3rd rib to the inframammary fold, which is at about the point of the 6th or 7th rib, and crossways from the border of the sternum to the anterior axillary line. The mammary tissue also expands erratically into the axilla as the glandular tail of spence. The posterior surface of the breast rests on segment of the fasciae of the pectoralis major, rectus abdominis muscles, external abdominal oblique and serratus anterior.

## A global prospective

Globalization, which thus greatly characterizes our period, was primarily linked with commercial-related actions, consequently with ecological concerns, and especially in recent times with the dark truth of terrorism. Up till now the “globalization” of numerous human being actions, together with health care, has been departing on for several decades, enhancing in step with improvement in information machinery. Correctly or incorrectly, and whether planned or not, numerous clinicians in developing countries keep an eye on what Americans are achieving, or are alleged to be achieving, as “state of the art” and recent most excellent performance that ought to be copied. The actions reviewed and our current narration with transplantation of bone marrow recommends that present “standard of care” United States described interventions are inappropriate to global utilization. Unluckily, extensive deficiency of community wellbeing strategies to breast cancer has made understanding of such rights for females not viable. Impractical, deadly, and extremely expensive treatments for breast cancer cannot assist the huge number of females in the earth at danger for or who develop breast cancer [31].

## Types of breast cancer

According to site, it is divided into invasive and non-invasive breast cancers

### Non-invasive breast cancer

It is a cancer that has not extended away from the lobule or ducts where it situated [32]. An example of a kind of non-invasive breast cancer is ductal carcinoma in situ. Ductal carcinoma in situ appears when atypical cells develop within the milk ducts, however have not extended to close proximity of tissue or outside. The word “in situ” describes “in place.” Even though the atypical cells have not extended to tissues outer the lobules or ducts, they can progress and grow into invasive breast cancer. The normal background of every scientific unit is demonstrated and a biological understanding of the accessible information is presented. Lobular carcinoma in-situ is understood merely a risky sign moderately than a predecessor for the successive growth of invasive cancer, so that one time the judgment is made, additional operative involvement is avoidable and sequential follow-up only is suggested. The management of ductal carcinoma in-situ should be kept in mind that breast-preserving treatment is at the present considered best therapy of breast cancer, the illness we are attempting to stop [33]. The pitfalls of suggested management based on retrospective statistics are have been taken into account and the requirement to conduct clinical studies intended to establish the best possible beneficial treatment of non-invasive breast cancer is affirmed [34].

### Lobular carcinoma in situ (LCIS)

This type of breast cancer develops into breast lobules [35]. The breast cancer has not extended exterior to the lobules into the breast tissue [36]. Lobular carcinoma in situ is usually identified as non-invasive breast cancer [37].

### Ductal carcinoma in situ

It is the most general kind of non-invasive breast cancer, is limited to the breast duct. Example of ductal carcinoma in situ is ductal comedocarcinoma [38].

### Invasive breast cancer

It exists when abnormal cells from within the lobules or milk ducts split out into close proximity of breast tissue [39]. Cancer cells can pass through the breast to different parts of the body through immune system or the systemic circulation [40]. They may move early in the development when the tumor is a minute or afterward when the tumor is huge Invasive breast cancer is most occurring general carcinoma in females. The regions of elevated threat are the prosperous populations of Australia and Europe wherever 6% of females suffer from invasive breast cancer prior to 75 years of age. The prevalence of breast cancer enhances quickly with increasing age [41]. Invasive breast cancer that extends to different organs of the body is also recognized as metastatic breast cancer [42]. Most common organ to which these cells spread are brain, bones, lungs and liver. These cells once more segregate and expand irregularly and produce new cancers. The new forming cells are developing in different part of the body, it is still breast cancer [43].

### Infiltrating lobular carcinoma (ILC)

Infiltrating lobular carcinoma is also recognized as invasive lobular carcinoma. ILC originates in the milk glands (lobules) of the breast, but frequently extends to other areas of the body [44].

### Infiltrating ductal carcinoma

Infiltrating ductal carcinoma is also recognized as invasive ductal carcinoma. IDC originates in the milk ducts of the breast and extends to the duct wall, invading the breast fatty tissues and probably other parts of the body [45].

### Medullary carcinoma

Medullary carcinoma is an invasive breast cancer that designs a discrete margin normal tissue and medullary tissue [46].

### Mucinous carcinoma

It is recognized as *colloid carcinoma*, mutinous carcinoma is a uncommon breast cancer created by the mucus-forming cancer cells. Females with mutinous carcinoma usually have an improved prediction than females with additional general kinds of invasive carcinoma [47].

### Tubular carcinoma

Tubular carcinomas are a particular kind of invasive breast carcinoma. Females with tubular carcinoma usually have an improved prospects than women with additional general kinds of invasive carcinoma [48].

### Inflammatory breast cancer

Inflammatory breast cancer is the form of swollen breasts (red and warm) with dimples and/or broad ridges due to cancer cells blocking lymph vessels or channels in the skin over the breast. Though inflammatory breast cancer is uncommon and is tremendously fast-growing [49]. Treatment involves vigilant synchronization of all multidisciplinary strategies, including radiation therapy, surgery, chemotherapy and imaging. The administration of neoadjuvant chemotherapy has accorded considerably to enhancement in general survival from the time when the earliest report of this matter and has performed the function of locoregional treatment such as radiation and surgery significant to sustained improvements in this ailment [50].

### Paget’s disease of the breast

It is the uncommon type of breast cancer that usually shows visible changes to the nipple of the breast [51]. Its symptoms include red itchy rashes involving the nipple and then it can sometime spread to the normal skin as well. However it resembles with the other skin conditions such as eczema and psoriasis but it can be differentiated as the other skin conditions usually affects both the breasts and can start from the areola rather than the nipple of the breast however Paget’s disease of the breast most often affects only one breast and starts with the nipple of the breast instead of areola (breastcancercare.org.uk) Nearly 1–3% of all the breast cancers are Paget’s disease and can affect both men as well as women. The actual theory behind the pathogenesis or development of Paget’s disease of the breast isn’t clear yet however there are few theories supporting it’s pathogenesis. Their warning signs include bleeding and oozing of discharge from the nipple, flattening or inversion of nipple, lump found in the breast etc. It can be diagnosed by using punch biopsy. It’s prognosis is good if it remains within the nipple or in ducts of the breast [52].

### Phyllodes tumor

Phyllodes tumors are can be either benign or malignant [53]. Phyllodes tumors develop in the connective tissues of the breast and may be treated by surgical removal [54]. Phylloides tumors are extremely uncommon; fewer than 10 females die of this kind of breast cancer every year in the United States [55].

### Triple-negative breast cancer

Breast cancer is at the present extensively documented that is a heterogeneous disorder with special sub-forms, distinguished by means of their dissimilar clinico-pathological characteristics, prevision and responses to management. Triple-negative breast cancer is described by the deficiency of progesterone receptor, human epidermal growth factor receptor 2 and estrogen receptor expression [56]. This type is mainly destructive, commonly observed in premenopausal females, and is responsible for 10–15% of cases in white females, with a elevated occurrence [57].

#### Pathogenesis of breast cancer

The breast is a complex tubulo-alveolar organ fixed within an asymmetrical connective tissue [58], that go through a chain of alteration from child bearing age to senility. The changes seen with every menstrual cycle and pregnancy guided us to assume the occurrence of precursor cells in the mature tissue that is able of synthesizing novel duct-lobular units [59]. The typical breast architecture contains a stratified epithelium bordered by a basement membrane and fixed in a template of blood vessels, lymphatic and stromal cells [60]. In the usual breast, the stratified epithelium comprised of two dissimilar cell populations, myoepithelial and epithelial, which can be distinguished by way of immunohistochemical staining with antibodies against myosin and CK, correspondingly. It has been postulated that the creation of cellular heterogeneity in breast disorders depends on the primary developmental series of the usual breast. This heterogenicity of the breast carcinoma might happen from the neoplastic change of either myoepithelial or epithelial cell, or yet from a stem cell that has the ability to develop into myoepithelial or epithelial cells [61]. According to the oncology of breast cancer, neoplastic cells differ from the normal body cells. Normal tissues of the body have limited growth promotion and regulation which helps to keep the structure and functions of tissues usual. However, cancerous cells have prolonged and chronic proliferation without any external stimuli [62]. Cancer cells overcome the growth suppressor genes [63]. Breast cancer is a malignant disease that initiates in the breast cells. Like other malignant tumors, there are numerous causes that can increase the possibility of developing breast cancer. Injure to the deoxyribonucleic acid (DNA) and hereditary alteration can guide to breast cancer have been associated with the exposure of estrogen. Some patients inherit fault in the deoxyribonucleic acid (DNA) and genes like the P53, BRCA1 and BRCA2 among others. The patients with a family history of breast or ovarian cancer have possibility of developing breast cancer [64]. Neoplastic cells require considerable potential to multiply and convert into a massive tumor [65]. The immune system usually tries to find out cancer cells and cells with injured deoxyribonucleic acid (DNA) and demolish them. Breast cancer might be outcome of malfunction of such an useful immune defence and surveillance. Breast cancer commonly occurs due to an association between genetic and environmental factors. RAS/MEK/ERK pathway and PI3K/AKT pathway defend normal cells from cell suicide. When mutation occurs in genes that are involved in encoding of these protective pathways, the cells become unable of committing suicide when they are no longer required which then leads to development of cancer. These mutations were confirmed to be experimentally associated with estrogen exposure [66]. It was recommended that deformity in the growth factors signaling can assist growth of malignant cells. Over expression of leptinin breast adipose tissue enhances proliferation of cell and cancer [67]. These are numerous growth factors signaling and other factors that interrelate between epithelial cells and stromal cells. Interruption in these might result in development of breast cancer. In cancer cells, enzyme telomerase turns away the chromosomal shortening and allows the extensive replication of cells [68]. Tumor cells get their nutrients and oxygen supply by angiogenesis [69]. Cancer cells break their boundaries and can enter into the blood, lymphatic tissues and other tissues of the body to produce a secondary tumor [70].

#### Causative factors and associated risks of breast carcinoma

Breast cancer is the leading cause of death among British females who are 40–55 years of age. Breast cancer is more common in single women than in married women [71, 72]. The breast is an estrogen sensitive organ. Many females who have been on birth control pills or estrogen replacement have found that the medications result in enlarged and often tender breasts. The activity of this medication, combined with the standard western high fat, low fiber diet, which over-stimulates breast tissue, could be a trigger for breast cancer. Incidence of breast cancer is higher in women above 50 years of age and 2 cases per 1000 are reported in this age group. Epidemiological investigations have also suggested that those women who have many children possess lower risk of breast cancer than those women who have fewer children. Incidence of breast cancer is 10.04% among all cancers and, most commonly occurs in 40–50 aged women. Mean age of breast cancer in Iranian women is 48 years [73]. An association of clinic pathological characteristic and breast cancer subtypes has been determined in Iranian women [74]. Breast cancer mostly occurs in obese women [75]. Depression is most commonly found in women with breast cancer [76]. Andsoy et al. [77] conducted a study to investigate knowledge of colorectal, cervical and breast cancer. For this study 226 working nurses were screened. It is very interesting that certain epidemiological studies have claimed that women who give birth to their child before they attain the age of 20 years are known to have decreased risk of breast cancer than those women who have not given birth to any child or who have given birth to their first child after the age of 30 years. The risk increases with age rapidly during premenopausal and slowly during post-menopausal life. Breast feeding decreases the risk of breast cancer [78]. Menopause resulting from surgical removal of ovaries (oophorectomy) decreases the risk [79]. Presence of certain kinds of benign tumours in breast increases the risk of malignancy [80]. The ovaries stop producing the female hormones once the menopause sets in, but in obese women the fatty tissue can provide the estrogen as it is capable of producing it. This increase in hormone production seems to increase the risk of breast cancer in obese post menopausal women. Deficiency of vitamin D and lack of sun exposure is considered to be the important cause of breast cancer [81]. It is found to be more in women than men [82]. The risk of breast cancer increases with age however rarely found before the age of 20 years [83]. Carcinoma in one breast can increase the risk by four times in another breast. While the patients that have the history of ovarian, endometrial or colon cancer have 1–2 times increased risk to develop breast carcinoma [84]. A female who has had breast cancer has an enhanced danger of occurring breast cancer in the other breast [85]. The minimal role of the gene has been established in the development of breast cancer. BRCA-1 (breast cancer susceptibility gene) is considered to be the cause of 5–10% of breast cancer that is transferred from either father or mother to the next generation. The study indicates that right environmental conditions are required for cancer promoting gene for expression. Certain families have been recognized with a genetically higher incidence of early onset breast cancer. If some individuals of the female’s family have had a specific kind of cancer, female may have an enhanced danger risk of breast cancer [86]. The danger is maximum if the affected family member had breast cancer at a juvenile period, had cancer in both breasts, or if female is a close family member. First-degree family members such as daughter, sister and mother are mainly significant in estimating threat. Numerous second-degree relatives such as an aunt and grandmother with breast cancer might also enhance threat. Breast cancer in a male enhances the danger for the entire close female relatives. Women who have a positive family history of breast carcinoma are 2–4 times more likely to develop the cancer, especially the females who are the carriers of BRCA1 or BRCA2 genes have the significant chance to develop carcinoma of breast [87]. Breast cancer affects both male and females; though, the prevalence is more in female as compared to male. Generally, females are at 100-fold increased danger of breast cancer than male [88]. Early menarche, nulli parity, pregnancy after the age of 30, oral contraceptives or hormone replacement therapy all these factors can increase the risk of breast cancer [83]. Steroid hormones include androgens, progesterone and estrogen, which belong to a cluster of structurally connected hormones known as sex hormones that are released into the blood by the gonads and adrenal glands. They are synthesized from single general precursor, cholesterol through a reaction catalyzed by numerous enzymes to make a large diversity of hormones for diverse target organs and tissues [89]. This procedure is well regulated and the discharge of these hormones into the systemic circulation. These hormones cross the plasma membrane to reach the target cells and bind to specific receptors called steroid hormone receptors to exert their activity [90]. Oestrogens have important activities on differentiation, growth and performance of several tissues, including urogenital system of man and women, cardiovascular system, brain, uterus and breast [89]. In accordance with this, Kato et al. [91], reported that the progression of reproductive organ cancer like prostate and breast cancer frequently occurs because of the androgens, progesterone and estrogen, which exert numerous biological activities in normal as well as abnormal cells. The study indicates that the development of normal and abnormal epithelial cells of the breast can be modulated by stromal cells of the breast and can release growth factors after stimulation by the endogenous hormones. An aromatase enzyme is found in adipose tissues, which makes estradiol from the precursor molecule, cholesterol. Fat cells are found in excess amount in breast of aged females; therefore, the quantity of estradiol is higher in breast tissues of post menopausal female than their plasma level [92]. This most likely is responsible for the increasing occurrence of breast cancer in aged female and assists the action of steroid hormones in breast cancer pathogenesis. Benign tumors and proliferative lesions without or with atypia can increase the risk of breast cancer [85]. Breast cancer has been linked with high level of dietary fats and low level of certain nutrients for various years [93]. Animal fat stimulates colonal bacterial to form estrogen from cholesterol found in the diet, thus increasing level of estrogen in the body. The body fat is also involved in synthesis of oestrone, a type of estrogen. Obesity, increased fat consumption, radiation therapy [94]. Evidence is accumulating that certain environmental pollutants contribute to estrogenic activity and may contribute to the prevalence of breast problems in the industrialized world. Alcohol consumption is linked with breast cancer risk. This association was felt to be secondary to the fact that consumption of alcohol enhances level of hormones in the blood [95].

### BRCA1 and BRCA2

These are two genes that have been recognized as possessing the firm relationship with breast cancer. Both emerge to contain comparable biological activities such as DNA damage repair and, in their variant kind, they enhance the danger of breast cancer and other ovarian cancers. The preeminent knowledge accessible to date is based on a joint investigation of 22 researches, 11 which established that the prevalence of breast cancer is 65% at 70 years of age in women who acquired a BRCA1 gene and 45% in those women who are carriers of BRCA2 genes. The prevalence of breast cancer in carrier of these genes is 10–30 times more than those females that have no inherited gene variants. In spite of the big boost in breast cancer danger linked with BRCA1 and BRCA2 genes, they are responsible approximately 5% of all breast cancers, as merely 1 in 1000 females have acquired one of them. There are several tribal Subgroups in which BRCA1 and BRCA2 genes are more possible to be innate (e.g. approximately 1% of females of Ashkenazi Jewish descent have acquired increased risk BRCA1 or BRCA2 genes; analogous variants are moreover familiar in women belong to Iceland and different Scandinavian countries). Individual risk indicator as an outcome of hereditary testing for BRCA1 and BRCA2 remains a demanding experience. Almost 2000 variants have been found in the two genes (BRCA1 and BRCA2) and, for numerous; it is yet not recognized whether or not they enhance prevalence of breast cancer. One possible sign is the site of the variant in the gene; it indicates that variants in few parts of the genes might cause bigger threat of breast cancer than others [96, 97]. Even though all females who acquire a BRCA1 or BRCA2 variant do not essentially cause breast cancer, it is uncertain what other determinants such as genetic or environmental persuade the risk of breast. There is also promising data that determinants for breast cancer might perform in a different way for carriers of BRCA1 or BRCA2 variants than for females lacking hereditary vulnerability because of these genes [98]. Literature review also recommends that high-risk genes other than BRCA1 and BRCA2 possibly enhance the risk of developing breast cancer, mainly for younger females.

### Mortality

Fifth most common cause of cancer death is breast cancer. The mortality and age standardized prevalence of breast cancer is higher in the United States compared to world. In Poland, seventeen percent of disease cases occur due to cancer and 14% deaths occur due to cancerous changes. Worldwide death due to breast cancer calculated in 2004 was 519,000 [99]. In the United States alone, approximately 1,208,000 cancer cases are reported per year and that some 538,000 people die from the previous manifestation of this disease condition, representing about one fifth of the total annual deaths from all causes [74].

### Stages of breast cancer

According to the report of breast cancer.org Stages of the breast cancer depends upon the size and type of tumor and how much the tumor cells have been penetrated in the breast tissues [100]. Whereas stage 0 describes the non invasive and stage 4 describes the invasive kind of tumor. Descriptions of those tumor stages are:

#### Stage 0

This is the non invasive stage of tumour which indicates that both cancerous and non cancerous cells are within the boundaries of that part of the breast in which the tumor begins to grow and no evidence found of their invasion in the surrounding tissues of that part, the example of this tumour stage is ductal cell carcinoma in situ (DCIS) [101].

#### Stage 1

This stage describes as the invasive breast carcinoma and microscopic invasion is possible in this stage. It has two categories that are 1A and 1B stage. The category 1A describes the tumor which measures up to 2 cm and none of the lymph nodes are involved in it while stage 1B describes that small group of cancer cells larger than 0.2 mm founds in lymph node [102].

#### Stage 2

Stage 2 also has two categories 2A and 2B. Stage 2A describes that the tumour is found in axillary lymph nodes or in sentinel lymph nodes but no tumor found in breast. The tumor can be smaller or larger than 2 cm but not more than 5 cm. However stage 2B describes that the tumor could be larger than 5 cm but can’t reach to the axillary lymph nodes [103].

#### Stage 3

It has been divided into three sub categories that are 3A, 3B and 3C. Amongst which stage 3A describes that no tumor is found in breast but it can be found in 4–9 axillary lymph nodes or in sentinel lymph nodes while stage 3B describes that the tumour can be of any size but have caused swelling or ulcer on the skin of the breast and can have spread up to 9 axillary lymph nodes or to sentinel lymph nodes stage 3B can be considered as inflammatory breast cancer which includes red, warm and swollen skin of the breast. However stage 3C describes the spread of tumor up to 10 or more than 10 axillary lymph nodes and it also have involved the lymph nodes above and below the clavicle [104].

#### Stage 4

This is the advanced and metastatic stage of cancer and this stage describes the spread to other organs of the body that is lungs, bones, liver brain etc [105].

## Diagnosis

### History and physical examination

The clinical history of patients with breast cancer is aimed at investigating cancer threat and demonstrating the occurrence or lack of manifestations indicative of breast illness [106]. It must comprise age at menarche, menopausal condition, earlier pregnancies and utilization of hormone replacement therapy after menopause or utilization of oral contraceptives. Personal history as well as family history should be carried out in detail. Personal history includes age at diagnosis of breast cancer, previous breast biopsies and treatment of other cancer with use of radiations. Family history includes history of ovarian cancers and breast cancer in first degree relatives. Patents should be examined for particular manifestations such as breast pain, weight loss, pain in bone, tiredness and nipple discharge [107]. Physical examination includes inspection of breasts, area around neck and collarbone, and armpits (axillae) carried out by clinicians [108]. Breasts are observed for any deformities such as lumps or other manifestations of breast cancer. Lymph nodes are also examined that are usually enlarged in patients with breast cancer.

### Self examination

Usefulness of the breast self-examination is contentious because the advantage in conditions of reduced deaths has not been established [109]. Most physicians educate females to carry out monthly BSE to become recognizable with their usual structure and authorize them with reference to their own healthcare [110]. Women are guided for self examination of the breast cancer. Women can find abnormalities in size and shape of breast on self examination [111–113]. Alipour et al. [114] conducted a study to investigate the the SMS based and paper based paper learner’s satisfaction and learning effect. Gynecologists gave printed materials and text messages regarding facts of breast cancer and breast cancer tests. Doctors found higher motivation and better effects in the SMS group than the printed material group. Sreedharan et al. conducted a study in United Arab States hospitals. Self administered structured questionnaire was used to investigate practices of self examination and knowledge. Satisfactory results were found from this study [115]. Ozkan et al. [116] investigated the level of knowledge regarding self examination of the breast cancer among 113 midwifery and nursing students. These researches have shown that continuous education program about breast cancer can raise the awareness among the population. Ceber et al. [117] conducted studies on breast self examination and health beliefs of Turkish women and stated that physical illnesses and early death can be prevented by early diagnosis of breast cancer. He further stated that one out of seven patients with breast cancer is diagnosed in time. Beydag and Karaoglan [118] investigated the awareness about breast self examination in 1st and 4th years students and concluded that 4th years students have more knowledge about breast examination than the 1st years students.

### Ultrasound breast imaging

There are numerous researches behind the application of adjunctive screening ultrasound in elevated hazard women with thick breast tissue, which reveals a significant but established figure of false positives [119, 120]. There is no randomized clinical study conducted for investigation of impact of screening ultrasonography on mortality rates of breast cancer. Entire breast ultrasound might permit the Physicians to display for breast tumors not measured by long-established mammography, particularly in thick breasts wherever mammography sensitivity is lesser [121]. Ultrasound breast imaging shows the size and position of tumour whether it is filled with fluid or is solid and needs to be biopsied to rule out cancer. This examination is quickly becoming a routine procedure for diagnosing lumps in young women [122, 123].

### Nuclear medicine

It is a type of molecular imaging wherever a radioactive substance (radiopharmaceutical) is introduced to an individual and the radiation from the radiopharmaceutical is displayed by perceptive emission detectors including gamma cameras and PET detectors and gamma Cameras located exterior to the body of patient. Combination of CT and gamma camera and the combination of CT and PET is a main progress in enhancing recognition and vicinity of disease.

### Single photon emission computerised tomography (SPECT)

This procedure utilizes solitary photon radionuclides including gallium-67, iodine-131 and technicium-99 m that discharge gamma rays. It is a efficient scan and is precise for organ of curiosity. It can also be employed to the entire body, is comparatively secure in expressions of radiation quantity and is fine in recognition of primary and metastatic cancers. Iodine-131 is together indicative and remedial for cancer of thyroid [124].

### Positron emission tomography (PET/CT)

In expressions of radiation quantity, PET/CT is also comparatively secure and utilizes positron emitting radionuclides including oxygen-15, flouoride-18 and carbon-11. The frequently utilized tracer in positron emission tomography is a radioactive type of glucose such as [18F]fluoro-2-deoxy-d-glucose. Tissues with enhanced metabolic requirements including developing cancer cells, demonstrate increased uptake of the tracer and displays on the scan. With combination of CT and PET, significant information regarding numerous situations affecting the different organs of the body is simply mapped. PET/CT is extremely perceptive and precise for predicating occult and different areas of loco-regional lymph nodal extent and/or far-away metastases not obvious by regular imaging, therefore altering staging in up to 25% of the patients. This procedure is employed for the management planning by describing spread of primary illness. It is also employed in re-staging after management ailment relapse and treatment follow up [125].

### Tumor markers

Porika et al. [126] stated that tumour markers should be measured in all stages of the breast cancer including metastasis prediction, treatment, diagnosis and screening. Thirteen verities of tumor markers of breast cancer are measured, six out of 13 are novel for the guideline. The different varieties displayed proof of clinical use and are suggested for utilization in practice [127]. It is particularly significant that the comparative autonomy of the markers in reference to other accessible markers to demonstrated so as to evade the gratuitous price and expenditure of redundancy [128]. Furthermore, it is significant that the physician be attentive of the restrictions in together specificity and sensitivity of every marker so because not to specificity and sensitivity of every marker so since not to over- or under-interpret the prognostic worth of a few investigation. With these caveats in intelligence, trial submission of tissue, germ-line and soluble tumor markers can recover medical care of individuals at threat for and with breast cancer.

### Ca 15-3

It can be employed for monitoring of patients with breast cancer. High blood levels are seen in <10% of patients in the beginning of breast cancer and in approximately 70% of patients with advanced stage of breast cancer. The levels of CA 15-3 typically fall after successful management. However CA 15-3 can also be high in other types of cancers and in few non-cancerous conditions including hepatitis and benign breast conditions.

### Ca 27.29

It is another marker for monitoring of patients with breast cancer. This test does not seem to be any better to identify early or advanced stage of breast cancer. This tumor marker is seen in other types of cancers and in few non-cancerous disorders.

### Estrogen and progesterone receptors

For the identification of breast cancer, breast cancer tissues are investigated for estrogen and progesterone receptors including HER2 antigen. These tests give information regarding the aggressiveness of cancer and response of certain drugs used for treatment of breast cancer.

### Immunohistochemistry

Immunohistochemistry (IHC) has grown to be an essential component of pathology. Although eosin and hematoxylin stain is the primary foundation for diagnostic pathology of the breast, Immunohistochemistry stains give valuable and sometimes very important information. Furthermore, taking into consideration the part of hormonal treatment in hormone receptor–positive breast tumors, as well as the accessibility of targeted chemotherapeutic drugs for HER2-positive patients, Immunohistochemistry knowledge indicates a key element of workups. Careful use of Immunohistochemistry stains in combination with E & H test assists determine mainly diagnostic matters encountered by clinicians during their routine practice. Clinicians should be well-known to utilize the each immunostain and its restrictions to evade errors in interpretation. Immunohistochemistry stains assists in differential diagnosis of challenging epithelial disorders of the breast. They should be selectively and sensibly utilized and their results must be understood with the differential diagnoses in consideration and with an understanding of potential drawback [129].

### MRI and breast cancer

Mammography has been considered as an appropriate screening method for breast cancer detection for many years [130] but it can’t distinguish between the solid and cystic masses and can miss up to 10–15% of the cases however MRI provides more accurate results and clear benefit to the women who are developing breast cancer due to the BRCA1 and BRAC2 genetic mutation and are present with the axillary lymph adenopathy [131].

### Breast biopsy

Breast biopsy is the simply best technique for diagnosing breast cancer [132]. There are numerous different types of breast biopsies. To enhance diagnostic precision and get rid of as many false negative results as possible, breast imaging, clinical breast examination and biopsy are performed concurrently (triple test).

### Fine needle aspiration

A thin prickle is employed to get cells from the abnormal area or a breast lump [133]. Ultrasound can be used to assist direct the prickle. A restricted anesthetic might be used to anesthetize the region where the prickle will be inserted [134].

### Core biopsy

A wider prickle is to get a portion of tissue (a core) from the abnormal area or breast lump [135]. It is typically made under restricted anesthetic, thus breast is insensitive, while patient may experience little hurt or uneasiness at what time the anesthetic is given [136]. MRI, ultrasound and mammogram can be used to guide the prickle for the duration of core biopsy [137].

### Vacuum-assisted stereotactic core biopsy

In this core biopsy, different tiny tissue samples are taken via single tiny incision in the skin with a prickle and a suction-type device [138]. It is carried out using local anaesthetic. MRI, ultrasound or a mammogram may be employed to direct the prickle into position. The patient may experience little uneasiness during the process [139].

### Surgical biopsy

If the abnormal vicinity is too minute to be biopsied by another procedure or the biopsy outcome is not apparent, a surgical biopsy is carried out. Prior to the biopsy, a guide wire may be placed into the breast to assist the medical doctor locate the abnormal tissue. Local anesthetic can be used and the physician may use MRI, ultrasound and mammogram to direct the wire into position. The biopsy is after that carried out under a general anesthetic. Little area close to breast tissue and lump are detached, alongside the wire [140, 141].

### Digital mammography

It helps to find lumps in dense tissue. The image can also be easily stored and transmitted to another radiologist for a second opinion [142–144]. Tarhan et al. [12] stated mammography may give false negative and false positive results in patients with dense breast tissues. Kanaga et al. [145] stated that the practice of mammography is 19% in Malaysian women as compared to other study which was 10.5%. Lack of health insurance coverage, low income and embracement were the main barriers to mammography as mentioned in earlier studies. Mammography is considered as the gold standard test for early detection of breast cancer [146] but in case of scarce resources in some areas in breast health awareness program should be promoted for the early detection of breast cancers and the staff should also gets the training of clinical breast examination so that the patient get diagnosed at earlier stage especially in those areas where mammography is unavailable [147].

### PEM and MRI in breast cancer patients

Hence both the positron emission mammography and magnetic resonance imaging have proven breast cancer detection sensitivity, however hormone replacement therapy, post menopausal status and breast tissue density has no influence on the sensitivity of PEM and MRI. Positron emission mammography can be used as an alternative of MRI in patients who don’t want to have an MRI due to multiple reasons such as time issues, limited budgets, lack of interest, claustrophobia (fear of being kept in as small space) [148]. However, both have the similar sensitivity to detect cancerous lesions comprehending invasive and ductal carcinoma in SITU [149].

### Treatment

In the management of breast cancer, aim is to preserve quality of life with prolonged life expectancy. The use of bioflavonoids may inhibit estrogen formation [150]. Effective communication between doctors and patients plays an important role to improve clinical outcome. Oshima et al. [151] reported that effective communication between doctors and patients is effective. A study conducted in Japan indicates that this communication helps the patients to cope with adverse effects. Doctor patient communication enhances the quality of life of breast cancer patients [152]. Previous studies have shown that less exposure from radiations, higher family monthly income, long years after diagnosis, higher education, initial stage cancer and younger age were considerably related with better quality of life (QOL) in patients with breast cancer [153]. Breast cancer is less common in breast feeding women, but the protective effect of this factor is not clearly investigated [154]. Cancer is a fatal disease affecting humankind in every country. Vinblastine and vincristine was introduced in 1961 as anti cancer drugs. CIPLA has improved the process of isolating vinblastine and vincristine in the World [155], and India is exporting these alkaloids to European countries and the demand is steadily increasing. The main forms of treatment for cancer in humans are surgery, radiation and chemotherapeutic agents. The drugs can often provide temporary relief of symptoms, lengthening of life and occasionally cures the disease. Many hundreds of chemical drugs of known classes of cancer chemotherapeutic agents have been synthesized [156]. The activity of these compounds is based on their capacity for biological alkylation. The effective dose of such alkylating agents was almost the same as the toxic dose. Multi-targeted therapy could be more effective, because the recurrence rate of cancer is high and death occurs due to metastasis. Deng et al. [157] reported that Pemetrexed and Lobaplatin is prescribed in metastatic breast cancer. Huang and Cao [158] reported that cantharidin sodium injection is effective in the management of breast cancer. Cantharidinate sodium injection is herbal origin and is prepared in China for treatment of breast cancer. Breast cancer management strategies differ depending on the step of the cancer—its mass, place, whether it has extended to other organs of the body and the physical condition of the individual. Present management for breast cancer includes targeted therapies, hormonal treatment, radiation therapy and surgery.

### Psychological adjustment to breast cancer

Breast cancer is extremely common and very worrying experience for numerous females every year in developing and developed countries [159]. Psychological research has given an image of the emotional and community impact of breast cancer on females’ lives, and of factors linked with better versus worse amendment. Psychosocial mediations have been helpful in reducing patients’ grief and improving their life quality. Current study also recommends that psychological aspects might be associated with potentially significant biological ailment linked processes. Additionally, to giving an idea of the psychological aspects in breast cancer, investigation in this vicinity has given a foundation for further studies on adjustment to health-related nervous tension in common [160].

### Surgery

This is the foremost management strategy for individuals whose breast cancer has not extended to further areas of the body and is also a choice for further complex stages of the illness [161–163]. The kinds of breast cancer surgery vary in the quantity of tissue that is excised with the cancer; this depends on the cancer’s characteristics, whether it has extended, and the patient’s special feelings. A few of the most familiar kinds of surgery include:

#### Lumpectomy (breast conserving surgery)

Some patients diagnosed with breast cancer undergo some type of surgery [164]. According to American cancer society, lumpectomy or partial mastectomy is the procedure of removing the part of the breast that contains malignant tumor along with some healthy tissues and surrounding lymph nodes leaving the major part of the breast intact as possible [165]. This practice generally experienced in women that are in their initial phase of cancer, however the patient also requires another type of treatment such as radiation therapy, chemotherapy or hormone replacement therapy along with this procedure. Most surgeons and patients prefer lumpectomy initially rather than having the complete breast removal, especially if the patient is more concerned about losing her breast [166]. However, adverse effects of lumpectomy are tenderness, temporary inflammation, sclerosis and changed appearance of breast, etc [167].

#### Mastectomy

Mastectomy is done to decrease the risk of development of breast cancer [168]. Bilateral prophylactic mastectomy decreases the chances of development of breast cancer but does not eliminate the risk of developing cancer completely [169]. Aromatase and tamoxifen decreases the risk of contra-lateral breast cancer and it is considered more effective than contra lateral prophylactic mastectomy [170]. Mastectomy is considered the most effective method of dealing with an already diffused case of breast cancer, for which a lumpectomy was not decisive enough. Nevertheless, the loss of breast leads to feeling of asexuality and loss of self-image and consequent depression in most women [171].

#### Reconstructive surgery

Females who have a mastectomy might as well have breast renovation, either immediate reconstruction or delayed reconstruction. It is performed to get better the look of the breast following tumor surgery. All females having a mastectomy must be presented the option to converse reconstructive surgical treatment [172]. Mastectomy is a comparatively simple surgical practice that typically results in stay in hospital for 1–2 days. Deficiency of the breast mass changes the patient’s special look and can create wearing a few forms of clothing difficult. The utilization of an exterior prosthesis to tackle these problems can be awkward and scratchy, particularly for females with huge breasts. Though, the most significant issue of mastectomy is the psychosocial effect of the physical and aesthetic distortion, which can comprise nervousness, sadness, and negative impacts on body figure and on sexual activity [173]. Breast reconstruction is commonly requested by females with breast cancer who are unable for breast-conserving treatment and females with an increased hereditary danger for breast cancer. Existing breast reconstruction procedures are miscellaneous and might engage the utilization of prosthetic implant or an autologous tissue flap, or both. Despite of the method employed, cancer might relapse in the reconstructed breast; additionally, in autologous tissue flaps reconstructed breasts, little complexity such as fat necrosis may take place. Researches recommend that breast reconstruction restores body representation, proves vigor, femaleness, and sexuality; and optimistically influences the patient’s feelings of comfort and life quality [174].

### Ovarian ablation as adjuvant therapy for breast cancer

Ovarian ablation has been employed as management for breast cancer [175]. There are numerous techniques of ovarian ablation such as radiation induced ablation, surgical removal of ovaries and chronic utilization of luteinizing hormone-releasing hormone (LHRH) analogs. Additionally, there are few proposals that cytotoxic chemotherapy might perform by inducing ovarian ablation in premenopausal females with breast cancer. Of the abundant case series and clinical studies of ovarian ablation conducted in the earlier period, numerous have been laden with methodologic issues. Meta-analysis of randomized clinical studies demonstrates a momentous enhancement in overall survival and disease-free survival for females whose ovarian ablation were performed as adjuvant treatment compared to those females who did not. Literature review indicates that ovarian ablation may be employed an alternative therapy for breast cancer [176].

### Role of estrogen and progesterone receptors in the management of breast cancer

The estrogen receptor assay has developed into a typical practice in the treatment of complex breast cancer [177]. Tumors missing estrogen receptor react occasionally to endocrine treatment, while improvement proportions of 50–60% are seen in estrogen receptor positive tumors. Current researches demonstrate that the estrogen receptor condition of the principal cancer is a superior interpreter of the endocrine reliance of metastatic cancers at the moment of clinical deterioration. Additionally, the deficiency of estrogen receptor in the primary cancer is an significant self-regulating predictive display of higher incidence of relapse and shorter survival. Quantitative investigation of estrogen receptor and an analysis for progesterone receptor are two procedures for enhancing the precision of selecting or rejecting individuals for hormonal treatment; cancers with a elevated quantitative estrogen receptor amount or those with a positive progesterone receptor show the maximum response. Initial investigation demonstrates that the existence of progesterone receptor might be a improved indicator of tumor hormone dependence than quantitative estrogen receptor [178].

#### Anti-estrogen therapy

It can be used in such types of cancers that are affected by hormones and the tumor has hormone receptors such as estrogen receptors. Clarke et al. [179] stated that the most common category of drugs that are used in breast cancer is anti estrogen, which includes the agents that are (tamoxifen, raloxifene, toremifene etc). Tamoxifen inhibits the hormone oestrogen from entering into cells of the breast cancer. This mechanism inhibits the breast cancer cells from developing. Tamoxifen can be suggested to treat female of any age group. However tamoxifen is considered as the drug of choice in women that have positive estrogen receptor breast carcinoma. Tamoxifen is a selective estrogen receptor modulator (SERMS) and acts like estrogen on other parts of the body such as uterus. However, it demonstrates anti estrogen properties of breast tissues and competes with estrogen for binding to the estrogen receptors in the breast [180]. If we have to discuss about the toxic effects of anti estrogen therapy, comparatively there is very least toxicity found in it as compared to other cytotoxic drugs [181]. While some patients withdraw the treatment before completing the course of drug due to the side effects such as hot flushes, gastro intestinal problems and vaginitis etc. Though, the medical indications for discontinuing antiestrogen therapy include adeno carcinoma, sarcoma and thrombo embolic diseases etc. Any how the American society of clinical oncology recommends Tamoxifen as standard adjuvant therapy for patients with Estrogen positive breast carcinoma [182]. On the other hand Fulvestrant; Faslodex has entirely anti estrogenic action and is considered as estrogen antagonist it demonstrates anti neo plastic activities in breast tissues without having a positive effect on the uterus and bones, which may lead to certain side effects if taken for a long period of time such as osteoporosis [183]. Tamoxifen and raloxifene are selective estrogen receptor modulators (SERMs), a set of medicine that selectively prevents or motivates oestrogen-like activity in different tissues, affecting the estrogen receptors [184]. Tamoxifen exhibits its oestrogen antagonist action in numerous tissues such as uterus, liver, bone and breast [185]. It was used as adjuvant treatment in estrogen receptor positive patients and tamoxifen was accepted by the United States Food and Drug Administration (FDA) in 1998 for the impediment of breast cancer for females at elevated danger [186]. This verdict was based on the outcome of a experiment carried out by the United States National Cancer Institute that was interrupted premature as an intervening study indicated that tamoxifen decreased breast cancer prevalence by approximately one half [187, 188]. Four big prospective studies have investigated the efficacy of tamoxifen versus placebo for breast cancer danger decline for females at elevated danger of breast cancer [189]. A summary of these studies demonstrated a 38% general decline in breast cancer occurrence for females at increased danger of breast cancer who administered tamoxifen for the period of 5 years and also indicated that tamoxifen inhibits only estrogen receptor positive breast cancers (RR ~ 50%) with no influence on estrogen receptor negative breast cancer [190]. A variety of adverse effects have been reported for females taking tamoxifen, such as venous thrombosis, cataract, endometrial cancer, menstrual disorders and hot flushes. A study indicated that the risk decreasing activity of tamoxifen expands beyond the vigorous management phase of 5 years, and remains for minimum 10 years, whereas the majority of adverse reactions do not carry on behind the 5 year management duration [191]. Raloxifene, has also been revealed to decrease danger of breast cancer, however seems to exert some adverse reactions [192]. During the precedent periods, clinical studies carried out to investigated the efficacy of raloxifen on fracture and osteoporosis, showed a 44–76% risk decline of breast cancer prevalence in the raloxifen treated patients as compared to the placebo group [193]. A randomized clinical study of Raloxifen and Tamoxifen was planned for comparing the efficacies of raloxifen and tamoxifen on postmenopausal females with an enhanced 5-year threat of breast cancer as expected by the Gail model [194, 189]. The study demonstrated that raloxifen was comparable to the tamoxifen in decreasing the threat of invasive breast cancer and was linked with a minor danger of cataract and thromboembolism than tamoxifen. In 2007, approximately 10 years following the endorsement of tamoxifen, the FDA permitted raloxifen for the impediment of breast cancer for postmenopausal females with osteoporosis and for postmenopausal females at increased danger for breast cancer. In Australia, tamoxifen is prescribed for the treatment of breast cancer and osteoporosis.

#### Aromatase inhibitors

These are compound designed for decreasing oestrogen formation by targeting aromatase, the enzyme complex accountable for the last stair in the formation of estrogen [195]. The third-generation aromatase inhibitors including letrozole, exemastane and anastrozole are in present utilization [196]. Randomized clinical trial conducted for investigation of these agents in the treatment of breast cancer has indicated that these compounds contain an outstanding effectiveness in treating females with advanced disorder. Clinical study indicated that females managed with aromatase inhibitors had a superior contra lateral breast cancer threat decline than females managed with tamoxifen [131].

#### Radiation therapy

It is useful for reducing the necessity of mastectomies. A combination of a lumpectomy and radiation therapy is being increasingly used over a mastectomy in the early stages of breast cancer [197]. A study was conducted in India. For this study 135 women were selected, most of them had undergone mastectomy. At the time of analysis, there was no local recurrence after hypo fractioned radiation therapy and metastatic disease developed in only four patients [198]. Zhou et al. [199] reported that radiation therapy is effective in early breast cancer patients. This study was conducted on 143 women who underwent either routine or intra operative radiation therapy after breast conserving surgery. At 54 months of follow up, there was a significant local control of the tumour. High-energy rays from radiation therapy kill cancer cells. This therapy affects only the cells that are treated. Use of radiation therapy may be done after breast cancer surgery to destroy the remaining cells in the chest area.

#### Brachytherapy

It **is** a kind of radiotherapy [200]. It might be recognized as accelerated partial breast irradiation. It directs radiation merely to the area around the vicinity wherever the cancer was. This might replace the requirement to provide radiation to the whole breast. It also decreases the number of management sessions [201].

#### Chemotherapy

The process of killing cancer cells by using certain medicines is termed as chemotherapy [202, 203]. It can be given in both situations, before and after surgery, depending upon the condition of the patient. According to the American cancer society the medicines include in chemotherapy are Docetaxel, Paclitaxel, Platinum agents (cisplatin, carboplatin), Vinorelbine (Navelbine), Capecitabine (Xeloda), Liposomal doxorubicin (Doxil), Cyclophosphamide (Cytoxan), Carboplatin (Paraplatin) etc [204]. However it has various side effects [205]. Metastatic or secondary breast can is difficult to treat but it can be controlled and sometime for various years [206]. Chemotherapy can be prescribed to manage metastatic breast cancer to minimize or sluggish its development. It can also be administered to decrease some manifestations. Other treatment option can be initiated prior or alongside chemotherapy.

#### Taxol

Taxol is used clinically in the treatment of ovarian cancers and is undergoing clinical trials against metastatic breast cancers [207]. It may also have potential value for lung, head and neck cancers. Taxotere is a side chain analogue of taxol, which has also been produced by semi synthesis from 10-deacetyl-baccatin III [208]. It has improved water solubility, and is being clinically tested against ovarian, and breast cancers. It can be used in those where resistance to cisplatin has been observed [209].

#### Anthracyclines

Anthracycline are commonly prescribed in the treatment of breast cancer [210]. They impede with enzymes associated the DNA copying, which is desired for cells to separate to create new cells. Epirubicin and doxorubicin are the most commonly used medicines in breast cancers. There is proof that anthracyclines functions better than various other chemotherapy medicines [211]. However these have adverse reactions such as damage to the heart and loss of hair [212, 213]. Prior to start of medicines, patient should converse with clinician any probable adverse reactions of drugs used and how these medicines might influence life quality.

#### Thermochemotherapy

Medifocus heat management in combination with chemotherapy enhanced the shrinkage of median cancer in the thermochemotherapy arm to 88.4%, whereas for chemotherapy alone the shrinkage of median cancer was 58.8%. For the thermo-chemotherapy management arm, approximately 80% of breast cancers had a cancer size decrease of 80% or more, compared to merely 20% for the chemotherapy alone [214].

#### Complementary therapies

Women with breast cancer occasionally desire to use complementary therapies along with their medical therapy [215]. These therapies are usually not investigated in randomized clinical trials [216]. Some female believe that they have benefited from a number of these treatments [217]. Vitamins, nutritional supplements, yoga, meditation, visualization, traditional medicines and acupuncture are included in complementary therapies.

## Medicinal plants

### Medicinal plants

Screening of plant extracts for anticancer activity started in 1961 by National cancer institute in the USA, and up to 1981 (20 years) about 1,14,045 plants had been screened of which only 3.4% (representing about 3400 different species) have been observed to be active in one or more biological systems.

#### *Ganoderma lucidum* (Polyporaceae)

It contains ganoderic acid, ganoderic acid G, ergosta, ergosterol peroxide ganoderic acid G, ergosta, ergosterol peroxide, methyl ganoderate A, B, ganoderic acid C2. It is an anticancer [218]. Jiang et al. [219] reported that the *Ganoderma lucidum* suppresses growth of breast cancer cells through the inhibition of Akt/NF-kappa B signaling. It is used to treat cancer cells. It inhibits the transcription factor NF-kappa B and inhibits the invasive behavior of breast cancer cells. The exact mechanism for inhibition of cancer cells is not understood. The study showed that the proliferation of breast cancer MDA-MB-231 cells is inhibited and Akt/NF-kappa B signaling is suppressed. Phosphorylation of Akt at Ser473 is suppressed by this plant and expression of Akt is suppressed, as a result NF-kappa B activity in MDA-MB-231 cells is inhibited.

#### *Momordica charantia* (Cucurbitaceae)

The parts used are fruits, leaves and seeds. It contains glucoside, albuminoids, fatty acids, non polar lipid, linolinic acid, palmitic acid, myrtenol, hexenol, benzyl alcohol, acylglycosylsterols and glycoproteins [220]. It is hepatoprotective, tonic, stimulant, emetic, laxative, stomachic and cancer [221]. It is used to treat gout and rheumatism. Ray et al. [222] reported that *Momordica charantia* extract inhibits breast cancer by modulating cell cycle regulatory genes. This study was conducted in vitro models. An extract of this plant was investigated in human breast cancer cells, MCF-7 and MDA-MB-231, and primary human mammary epithelial cells. This extract was able to decrease cell proliferation and apoptotic cell death was induced. Survivin and claspin expression was inhibited by this extract.

#### *Carthamus tinctorius* (Asteraceae)

The parts used are flowers and seeds. It contains palmitic acid, hexadecanolenin, coumaric acid, daucosterol, apigenin, kaempferol, trans-3-tridecene-5, 7, 9, 11-tetrayne-1, 2-diol, trans-trans-3, 11-tridecadiene -5, 7, 9-triyne -1, 2-diol [223]. It is used in colds, flu, fevers, hysteria, anemia, and diabetes mellitus. It is an antioxidant [224] and alpha glucosidase inhibitor [225]. Loo et al. [226] reported the efficacy of this plant in breast cancer. MDA-MB-231 breast cancer cell and normal human mammary gland cell were treated with a compound that contains Carthamus tinctorius. This compound observed inhibition of cell proliferation. Inhibition of cell proliferation was dose dependent. Its cytotoxic activity was more than commonly used cytotoxic drugs.

#### *Viscum album* (Viscaceae)

Part used are leaves and stem. It contains sinapylflavanone, glucopyranoside, flavanone, hydroxy flavanone and viscin [227]. It is antioxidant, cardiac tonic, and anti-cancer [228]. It is used in palpitation, vascular spasms, asthma, dizziness, vertigo and headaches. Gunver et al. [229] reported the efficacy of this plant in breast cancer.

#### *Calendula officinalis* (Asteraceae)

The parts used are leaves. It contains triterpene, calendula glycoside, butyl ester, flavonol glycosides, and carotenoids [230]. It is anti-inflammatory and anti-cancer [231]. It is used in carcinoma of the vagina, and cervix. Pommier et al. [232] reported the efficacy of *Calendula officinalis* for the prevention of acute dermatitis during irradiation for breast cancer.

#### *Citrullus colocynthis* (Cucurbitaceae)

The parts used are seeds and fruit. It contains phytosterol, flavones C-glycosides, saponins, aspartic acid, arginine, colocynthin, colocynthitin and cucurbitacin glycosides [233]. It is used in constipation and carcinoma of the breast [234]. It is an emmenagogue, ecbolic, cathartic, hydragogue and antioxidant [235]. This plant has growth inhibitory activity. Cucurbitacin glucosides have been isolated from this plant. These glycosides prevent human breast cancer cells [234].

### Indole-3-Carbinol (13C)

A compound known as indole-3-carbinol, which is a plant chemical derived from cruciferous vegetables such as Brussels sprouts and cabbage, changes the way estrogen is metabolized. This compound predictably alters the endogenous estrogen metabolism towards increased catechol estrogen production and may thereby provide a novel dietary means for decreasing risk of breast cancer [236].

### Silibinin and Chrysin

Previous research indicates that chrysin and silibinin function synergistically and possess significant anti-cancer activities against T47D breast cells [187, 188]. It shows potential that the synergistic efficacy is based, at least in part, by down-regulation of hTERT and cyclin D1. Their potential activities in the established synergism among Chrysin and Silibinin should be verified by additional in vitro or in vivo researches. Study demonstrates that Chrysin and Silibinin combined might come out as an eye-catching approach based on herbal medicine for the management of breast cancer [237].

### *Lactobacillus acidophilus*

Breast cancer and hyperestrogenism may be decreased by the inclusion of lactobacillus acidophilus in the diet. This useful bacterium helps to metabolize estrogen properly in the bowel. Clinicians can prescribe lactobacillus acidophilus that is available in different forms, including capsules in patients with breast cancer [238].

### Selenium

Women with breast cancer have been shown to possess selenium levels that are lower than those of women without cancer. Selenium is a trace mineral that is often lacking in refined food diets. A contrary association exists among the prevalence of human breast cancer and concentration of dietary selenium. The adding of Selenium to the food has been revealed to reduce the occurrence of breast cancer [239].

### Targeted therapies

These are drugs prescribed to manage some types of breast cancer. The mainly familiar targeted treatment is the drug Herceptin [240]. It is prescribed to manage HER2 positive breast cancer. It functions by preventing the cancer cells from developing and progressing [241].

### Gene therapy for carcinoma of the breast

Gene therapy is a remedial strategy that is considered to correct particular molecular deformities associated with the progression or development of breast cancer [242]. Mutated BRCA1 and p53 genes recognized as cancer susceptibility gene are involved in progression of cancer [243]. Since mutational inactivation of gene activity is reserved to cancer cells in these contexts, cancer gene modification techniques may give an opening for selective targeting without major hazards of normal, non-cancer cells [244, 245]. Both BRCA1 and p53 emerge to restrain tumor cells that lack mutations in these genes, indicating that the so-called gene modification techniques may contain broader efficacy than previously considered. Raising awareness of cancer genetics has recognized these and new genes as possible targets for gene substitute treatment [246]. Early patient study of BRCA1 and p53 gene therapy have given a number of indications of possible effectiveness, but have also recognized areas of clinical trials that are wanted prior to these therapeutic strategies may be broadly employed in patients with breast cancer [247].

### Oncogenes inactivation

Numerous oncogenic proteins have been recognized and linked with a variety of cancers [248]. The frequently practical strategy in clinical studies is the employment of antisense options. Oncogenes transcription also can be prevented by means of adenoviral gene E1A, which hinder erbB-2 transcription, an option helpful in managing cancer that over express this oncogenic protein [249].

### Augmentation of cancer suppressor genes

The mutations in tumor suppressor genes are linked with the development of numerous cancers. Some clinical studies are being conducted to deliver p53 via adenoviral vectors to different cancers. Likewise, viral vectors have been used to administer a breast cancer gene BRCA1 and retinoblastoma gene into ovarian cancer and bladder, correspondingly. In various circumstances, this strategy will fall short, as the mutant gene indicates dominant negative activity of the normal gene. To avoid this difficulty for p53 gene therapy, a genetic repair approach rather than a gene augmentation strategy might be more successful [250].

### Cancer stem-cell therapy for breast cancer

Current investigation in biology of breast has provided the foundation for the cancer stem-cell hypothesis [251]. Two significant aspects of this theory are that cancer arises in progenitor cells or mammary stem cells as an outcome of dysregulation of the normally strongly regulated method of self-renewal. As a consequence, cancers posses and are obtained by a cellular subcomponent that keeps central stem-cell functions such as self-renewal, which directs differentiation and tumorigenesis that is responsible for cellular heterogeneity. Development in the stem-cell field have guided to the recognition of stem cells in normal and malignant tissue of the breast. The investigations of these stem cells have assisted to clarify the source of the molecular complexity of breast cancer in human. The cancer stem-cell theory has significant role for timely recognition, prevention, and management of human breast cancer. Dysregulation of stem cell renewal pathways are involved in the development of both sporadic and hereditary breast cancers. These abnormal stem cells may give targets for the improvement of cancer prevention options. In addition, since breast cancer stem cells may be extremely challenging to chemotherapy and radiation, the progress of additional efficient treatments for breast cancer may need the efficient targeting of this cell population [252].

### Anti-oestrogens and prevention of breast cancer

With the accomplishment of anti-oestrogens in breast tumor management, numerous studies evaluated their use as an mediator to avert breast cancer in female at high risk [253, 254]. Tamoxifen is the antiestrogen medicine employed most commonly in the treatment of breast cancer. Administration of tamoxifen as an adjuvant treatment following surgery, normally for 5 years, decreases the risk of hormone receptor breast cancer recurrence.

Metastatic breast cancer is also managed by tamoxifen. In numerous females, tamoxifen induce the manifestations of menopause such as mood swings, vaginal discharge and hot flushes. Toremifene is one more medicine strongly related to tamoxifen. It is used an alternate drug in postmenopausal female for the treatment of metastatic breast cancer. Fulvestrant is another drug that decreases the estrogen receptor numbers. It is usually useful in postmenopausal female, even in tamoxifen resistant breast cancer. In previous studies, tamoxifen was evaluated for its efficacy in 13, 388 females at higher risk of breast cancer for the period of 5 years. The study indicated a 49% decrease in risk of increasing invasive breast cancer and as well decreased risk of opposing side breast cancer, reappearance and extended existence in the female who had tamoxifen as accessory after operation [187, 188]. Antioestrogens are currently suggested as chemoprevention for female with atypical hyperplasia, genetic tendency to develop cancer and important family history of breast tumor. They are also prescribed because component of practice post-operative concomitant management of those with estrogen receptor positive cancers for duration of 5 years following surgery [255].

### Human monoclonal antibody

Monoclonal antibodies are prepared in the laboratory [256]. These are used alone or in combination with radiation therapy and chemotherapy to locate and target cancer cells. Usually, the body’s immune system attack to foreign antigens such as infectious agents. It will then create antibodies to assist fight it off. The body does not identify cancer cells as a kind of foreign attacker. So, antibodies are then not formed. A randomized clinical trial was conducted to investigate efficacy of denosumab, a completely human monoclonal antibody against receptor activator of nuclear factor κ B (RANK) ligand, in comparison with zoledronic acid in the prevention of skeletal-related events in breast cancers individuals with bone metastases. Denosumab was found better as compared to zoledronic acid in preventing or delaying the SREs in breast cancer patients with bone metastasis. It is demonstrated that denosumab is possible therapeutic alternative for individual with bone metastases [257].

### Immunotherapy

It utilizes the immune system of the body to fight against the cancer cells [258]. Cancer vaccine is one of its examples. Parts of cancer cells or cancer cells are utilized for formation of vaccines. These cells excite the body’s immune system to assist assault and destroy cancer cells [259]. Immunotherapy has turn into a significant constituent in the management of breast cancer. HER2 targeted treatment are at the present an important part of HER2 over expressing breast tumor therapy. Trastuzumab, with the new current accompaniments of pertuzumab and TDM1, encompass considerably superior breast cancer prediction. With various Federal Drug and Administration recommended antibody treatments used in together the adjuvant and metastatic settings, development progresses to be done in the area of immunotherapies. Current achievements in targeted therapies, vigorous particular immunotherapy, grasp assure for continuous success in general endurance within the adjuvant setting. The extremely precise and targeted strategy of vaccine therapy not simply avoids the adverse effects of recent standard of care therapies, active and passive immunotherapies including ipilimumab; however presents remedial strategy beyond now the HER2-overexpressing individuals. Even though vaccines for breast cancers have been mainly ineffective in precedent clinical studies, the most of these studies conducted in the location of late-stage metastatic illness, adverse surroundings for agents intended to stop, as different to manage, disease. With present clinical studies conducted on the adjuvant settings, immunogenicity is at the present indicating association with medical response.

### Anti–angiogenesis drugs

Angiogenesis and inflammation are host-dependent manifestations of tumors that can be targeted with impediment strategies long prior to cancer start and develop [260]. Numerous prescription and non-prescription medicines are now accessible for utilization in angioprevention. Angioprevention can be proposed at four levels; first for the healthy people, 2nd for population at enhanced risk of tumor, 3rd for preneoplastic disease and 4th for prevention of cancer relapse. There are numerous achievements in prevention of cancer that reveal medical possibility and levels of interference, from no to slight to strong clinician participation. To evade toxicity whereas maintaining effectiveness, angioprevention desires to attain a level of angiogenesis prevention that is not extremely oppressive, such that hale and hearty vascular activity is maintained. These drugs block angiogenesis. In the absence of blood supply to cancer cells, they cannot develop and die. Various drugs are under investigation for the management of metastatic breast cancer. In initial stage of breast cancer, they are also investigate in the neoadjuvant (before surgery) setting [261]. Antiangiogenic treatment in breast cancer presents important promise, and numerous continuing investigations are trying to better describe the best management settings and mediator assortment. For patients with estrogen receptor positive aliment, researches recommend a relationship among resistance of endocrine and cancer dependence on angiogenic networks, suggesting a possible curative advantage in mixing endocrine treatment with antiVEGF mediator. Findings from randomized clinical studies emphasize the multiplicity in reaction to antiVEGF treatment and recommend the requirement for better choice of patient subsets further to be expected to advantage from these therapies. The recognition of biomarkers for therapy response is solitary part of deep attention, though mainly study to date has become unsuccessful to discover a relationship linking cancer-associated markers including cancer mutations and EGF expression and scientific response.

### Surveillance and follow up

A regular assessment of the important in print literature conducted by de Bock et al. [262], revealed that 40% of recurring cancers are identified in asymptomatic individuals during routine visits. This information intensifies the significance of surveillance and follow-up. Clinical investigation such as history and physical examination is suggested each 4–6 months for 5 years, after that each year with annual mammography. Female on tamoxifen should go through a yearly gynecologic evaluation if the uterus exists. Female who suffers from ovarian failure secondary to management or on an aromatase inhibitor should have checking of bone fitness with a bone mineral thickness determination at the start and sometimes subsequently. Women should also be advised to adopt variable risk factors, including lessening alcohol use, reducing BMI and enhancing physical activity.

## Conclusion

The increase of information on the pathophysiologic mechanisms of breast cancer has brought extensive development in the figure of biomolecular markers. In addition, the development of targeted drug design has grown quickly and more complicated, providing numerous agents that target these markers for in vivo investigation in animal models as well as clinical studies. The enthusiasm among scientists and Physicians about the growing management strategies is tempered by apprehension that resources are insufficient to carry the mainstream of these agents to advanced clinical trials. The challenges, then, are to choose the most capable agents to be investigated and the proper clinical studies for such evaluations. We have adopted a justifying strategy to unfolding the most extensively documented molecular targets in breast cancer. Drugs that amend the NRF have not been evaluated comprehensively so far, and such studies can boost the chances for true ‘endocrine’ strategies for management of breast cancer. Furthermore, agents that amend angiogenesis and apoptosis demonstrate an thrilling area of research, mostly in vigilantly chosen combination regimens.
